# Fokus Neurologische Intensivmedizin: Intensivmedizinische Studien aus 2019/2020

**DOI:** 10.1007/s00101-020-00861-z

**Published:** 2020-10-13

**Authors:** D. Michalski, C. Jungk, T. Brenner, M. Dietrich, C. Nusshag, C. J. Reuß, M. O. Fiedler, M. Bernhard, C. Beynon, M. A. Weigand

**Affiliations:** 1 Klinik und Poliklinik für Neurologie, Universitätsklinikum Leipzig, Leipzig, Deutschland; 2 Neurochirurgische Klinik, Universitätsklinikum Heidelberg, Heidelberg, Deutschland; 3 Klinik für Anästhesiologie und Intensivmedizin, Universitätsklinikum Essen, Essen, Deutschland; 4 Klinik für Anästhesiologie, Universitätsklinikum Heidelberg, Heidelberg, Deutschland; 5 Klinik für Endokrinologie, Stoffwechsel und klinische Chemie/Sektion Nephrologie, Universitätsklinikum Heidelberg, Heidelberg, Deutschland; 6 Zentrale Notaufnahme, Universitätsklinikum Düsseldorf, Düsseldorf, Deutschland

Dieser Beitrag ist Teil einer Serie zu den wichtigsten intensivmedizinischen Studien aus 2019/2020. Alle Artikel stehen Ihnen auf www.springermedizin.de zur Verfügung. Bitte geben Sie dort den Beitragstitel (Fokus) in die Suche ein.

Die Serie umfasst:

- *Fokus Allgemeine Intensivmedizin:* Dietrich M, Beynon C, Fiedler MO et al (2020) Anaesthesist. 10.1007/s00101-020-00857-9
- *Fokus Beatmung, Sauerstofftherapie und Weaning:* Fiedler MO, Reuß CJ, Bernhard M et al (2020) 10.1007/s00101-020-00859-7
- *Fokus Neurochirurgische Intensivmedizin:* Beynon C, Bernhard M, Brenner T et al (20202) 10.1007/s00101-020-00858-8
- *Fokus Neurologische Intensivmedizin:* Michalski D, Jungk C, Brenner T et al (2020) 10.1007/s00101-020-00861-z
- *Fokus Nephrologie:* Nusshag C, Reuß CJ, Dietrich M et al (2020) 10.1007/s00101-020-00856-w

**Table 1:** 

Originaltitel der Studie	Ergebnis – Kurzzusammenfassung
Randomized trial of three anticonvulsant medications for status epilepticus (ESETT: Established status epilepticus treatment trial) [2]	In dieser randomisierten Studie unterschieden sich die Antiepileptika Valproat, Levetiracetam und Fosphenytoin (nur zugelassen in den USA) nicht hinsichtlich ihrer Wirksamkeit bei der Durchbrechung eines benzodiazepinrefraktären Status epilepticus, basierend auf klinischen Kriterien
Association of surgical hematoma evacuation vs. conservative treatment with functional outcome in patients with cerebellar intracerebral hemorrhage [9]	Unter Einschluss von 4 Beobachtungsstudien zeigte diese Metaanalyse in Bezug auf das funktionelle Behandlungsergebnis keinen Vorteil für eine chirurgische Therapie bei Patienten mit zerebellären Hirnblutungen gegenüber der konservativen Therapie, wenngleich die chirurgische Therapie mit einer größeren Wahrscheinlichkeit des Überlebens assoziiert war
Intravenous thrombolysis prior to mechanical thrombectomy in large vessel occlusions [13]	Bei der Behandlung des ischämischen Schlaganfalls mit vorliegendem Hauptstammverschluss zeigte diese Metaanalyse auf der Basis von 38 Beobachtungsstudien bzw. Post-hoc-Analysen einen Vorteil für das „Bridging“-Konzept, d. h. die kombinierte systemische Thrombolyse und mechanische Thrombektomie, gegenüber der alleinigen mechanischen Rekanalisation in Bezug auf das funktionelle Behandlungsergebnis
Association of general anesthesia vs. procedural sedation with functional outcome among patients with acute ischemic stroke undergoing thrombectomy: a systemic review and meta-analysis [16]	Unter Einschluss von 3 randomisierten klinischen Studien lieferte diese Metaanalyse einen Vorteil für die Allgemeinanästhesie gegenüber der alleinigen Sedierung während der mechanischen Thrombektomie in Bezug auf das funktionelle Behandlungsergebnis
Ventilation time and prognosis after stroke thrombectomy: the shorter, the better [17]	In dieser monozentrischen Kohortenstudie war eine Beatmungsdauer von mehr als 6 h im Rahmen der Allgemeinanästhesie, die während der mechanischen Thrombektomie zur Behandlung des ischämischen Schlaganfalls mit vorliegendem Hauptstammverschluss durchgeführt wurde, mit einem ungünstigeren funktionellen Behandlungsergebnis assoziiert
Blood pressure and outcome after mechanical thrombectomy with successful revascularization [20]	Diese multizentrische Kohortenstudie zeigte eine Assoziation zwischen dem funktionellen Behandlungsergebnis und einem erhöhten Blutdruck innerhalb der ersten 24 h bei Hirninfarktpatienten, die bei vorliegendem Hauptstammverschluss mit einer mechanischen Thrombektomie behandelt wurden, wobei mittlere systolische Werte von mehr als 140 mm Hg mit einem schlechteren Behandlungsergebnis vergesellschaftet waren

## Status epilepticus

### Originalpublikation

Kapur J, Elm J, Chamberlain JM et al (2019) Randomized trial of three anticonvulsant medications for status epilepticus. N Engl J Med 381:2103–2113

Die Empfehlungen der neurologischen Fachgesellschaft betonen die stadiengerechte Behandlung des Status epilepticus im Erwachsenenalter, bei der sich an die 1. Stufe mit bevorzugter i.v.-Benzodiazepin-Applikation die 2. Stufe mit Anwendung spezifischer antiepileptischer Medikamente, im Detail Phenytoin, Valproat, Levetiracetam oder Phenobarbital, anschließt [1]. Die potenzielle Überlegenheit einer dieser Substanzen gegenüber einer anderen der 2. Stufe war bisher in keiner randomisierten Studie untersucht worden, sodass sich die Wahl der zuerst zur Anwendung kommenden Substanz zumeist an den persönlichen Erfahrungen der Behandler und den Vorerkrankungen der Patienten orientierte.

In der im Jahr 2019 veröffentlichten, randomisierten, multizentrischen, verblindeten Studie untersuchten Kapur et al. [2] die Effektivität und Sicherheit von Valproat und Levetiracetam sowie dem in den USA, nicht aber in Deutschland zugelassenen Fosphenytoin, bei Kindern und Erwachsenen mit benzodiapezinrefraktärem konvulsivem Status epilepticus. Hierfür rekrutierten 57 Zentren in den USA im Zeitraum 2015–2017 insgesamt 384 Patienten mit relativ gut balancierten Behandlungsarmen (Valproat *n* = 121, Levetiracetam *n* = 145, Fosphenytoin *n* = 118). Primärer Endpunkt waren das Fehlen von anfallsverdächtigen Symptomen (v. a. Myoklonien und rhythmischen Augenbewegungen) und eine Verbesserung der Reaktionsfähigkeit bzw. Ansprechbarkeit 60 min nach Beginn der jeweiligen i.v.-Gabe. Im Ergebnis wurde der primäre Endpunkt bei 56 (46 %) der mit Valproat, bei 68 (47 %) der mit Levetiracetam und bei 53 (45 %) der mit Fosphenytoin behandelten Patienten erreicht. Die Überlegenheit einer Substanz konnte dabei nicht gezeigt werden. Hinsichtlich der Sicherheit der 3 Substanzen fanden sich keine statistisch signifikanten Unterschiede, bezogen auf die folgenden Parameter: lebensbedrohliche Kreislaufdepression, lebensbedrohliche kardiale Arrhythmien, respiratorische Insuffizienz, endotracheale Intubation, wiederkehrende Anfälle, akute anaphylaktische Reaktionen, akute Leberwert- oder Ammoniakerhöhungen, „Purple-glove“-Syndrom (eine für Phenytoin beschriebene Hautreaktion) und Tod. Rein numerisch waren jedoch mehrere dieser Parameter im Zusammenhang mit der Anwendung von Fosphenytoin erhöht, wie beispielsweise eine Kreislaufdepression bei 4 Patienten (3,2 %) im Vergleich zu 2 Patienten (1,6 %) nach der Valproat- und nur einem Patienten (0,7 %) nach der Levetiracetamgabe.

Zusammenfassend konnten Kapur et al. [2] eine vergleichbare Effektivität und Sicherheit der in der 2. Stufe der Statusbehandlung üblicherweise zum Einsatz kommenden Substanzen zeigen. Im Alltag scheint es daher weiter gerechtfertigt zu sein, bei der Auswahl des spezifischen Antiepileptikums patientenbezogene Faktoren in den Vordergrund zu rücken. Am bedeutendsten erscheint dabei die Berücksichtigung (mindestens relativer) Kontraindikationen wie Leberfunktionsstörungen und Thrombozytopenien bei der Anwendung von Valproat, einer Niereninsuffizienz bei der Anwendung von Levetiracetam sowie kardialer Vorerkrankungen und hier im besonderen atrioventrikulärer Überleitungsstörungen und des „Sick-sinus“-Syndroms bei der Anwendung von Phenytoin. Sich im Alltag ergebende Überlegungen – wie beispielsweise der theoretische Nutzen einer Kombination mehrerer spezifischer Antiepileptika gegenüber einem Präparatewechsel oder der mögliche pharmakologische Vorteil einer kontinuierlichen gegenüber einer diskontinuierlichen Gabe – sind in der Studie von Kapur et al. (2019) nicht adressiert worden und bedürfen weiterer Untersuchungen.

## Intrazerebrale Blutungen

### Originalpublikation

Kuramatsu JB, Biffi A, Gerner ST et al (2019) Association of surgical hematoma evacuation vs. conservative treatment with functional outcome in patients with cerebellar intracerebral hemorrhage. JAMA 322:1392–1403

Randomisierte klinische Studien zur Behandlung intrazerebraler Blutungen konnten bisher keine Überlegenheit einer frühen chirurgischen [3, 4], auch minimal-invasiven Therapie [5] gegenüber einem konservativen Vorgehen zeigen. Metaanalysen lieferten dennoch einen Trend hin zu einer reduzierten Sterblichkeit im Zusammenhang mit der chirurgischen Therapie bei bestimmten Blutungsmerkmalen [6], sodass diese im Einzelfall und oftmals mit der Intention eines lebensrettenden Eingriffs bei ausgeprägt raumforderndem Effekt zum Einsatz kommt [7, 8]. Eine besonders schlechte Datenlage existiert für infratentorielle Hirnblutungen, sodass diesbezügliche Empfehlungen im Vergleich zu supratentoriellen Blutungen noch schwieriger erscheinen.

In einer Metaanalyse untersuchten Kuramatsu et al. [9] den Effekt einer chirurgischen Therapie gegenüber dem konservativen Vorgehen bei Patienten mit infratentoriellen, genauer zerebellären Blutungen. Hierfür herangezogen wurden die Datensätze von 4 Beobachtungsstudien (RETRACE‑I, -II und UKER aus Deutschland, ERICH aus den USA) zu intrazerebralen Blutungen mit insgesamt 6580 Patienten, die im Zeitraum von 2006 bis 2015 behandelt wurden. Von diesen wiesen 578 Patienten eine zerebelläre Blutung auf, und wiederum 174 Patienten erhielten eine chirurgische Therapie in Form einer Hämatomausräumung. Nach einem aufwendigen „Propensity-score-matching“-Verfahren, das bereits in der ersten Stufe die Faktoren Alter, neurologische Beeinträchtigung (Glasgow-Koma-Skala), Blutungsvolumen und den intraventrikulären Blutungsanteil berücksichtigte, wurden 152 Patienten mit zerebellären Blutungen und einer erfolgten chirurgischen Therapie einem ebenso großen Patientenkollektiv mit konservativem Vorgehen gegenübergestellt. Als primärer Endpunkt diente der Anteil an Patienten mit gutem funktionellen Zustand nach 3 Monaten, definiert als modifizierte Rankin-Skala (mRS) 0–3. Infolge der chirurgischen Therapie wurde dieser Endpunkt von 47 Patienten (30,9 %) und nach der konservativen Therapie von 54 Patienten (35,5 %) erreicht („odds-ratio“ (OR) 0,94; Konfidenzintervall (KI) 0,81–1,09; *p* = 0,43). Somit zeigte sich kein Vorteil für die chirurgische Therapie, die jedoch mit einer größeren Wahrscheinlichkeit des Überlebens nach 3 Monaten (OR 1,25; KI 1,07–1,45; *p* = 0,005) und einem Jahr (OR 1,21; KI 1,03–1,42; *p* = 0,02) verbunden war. In weiterführenden Analysen fanden sich Hinweise auf eine maßgebliche Bedeutung des Blutungsvolumens. Im Detail war die chirurgische Therapie bei Patienten mit einem Blutungsvolumen ≥15 cm^3^ mit einer höheren Wahrscheinlichkeit für das Überleben assoziiert (*p* = 0,02).

Wenngleich randomisierte prospektive Studien speziell bei infratentoriellen Blutungen weiter ausstehen, liefert die Metaanalyse von Kuramatsu et al. [9] durch das Zusammenführen großer Studienpopulationen robuste Daten, die keinen Vorteil der chirurgischen Therapie in Bezug auf den funktionellen Zustand 3 Monate nach dem Ereignis nahelegen. Vergleichbar mit der Situation supratentorieller Blutungen, scheint sich die chirurgische Therapie bei Patienten mit größeren Blutungsvolumina vorteilhaft in Bezug auf das Überleben auszuwirken, sodass der Eingriff im Einzelfall als Ultima-Ratio-Ansatz diskutiert werden kann.

## Systemische Thrombolyse und endovaskuläre Therapie

### Originalpublikation

Katsanos AH, Malhotra K, Goyal N et al (2019) Intravenous thrombolysis prior to mechanical thrombectomy in large vessel occlusions. Ann Neurol 86:395–406

Dass endovaskuläre Therapieverfahren wie die mechanische bzw. Aspirationsthrombektomie bei akutem Hirninfarkt mit zugrunde liegendem Hauptstammverschluss (im Englischen: „large vessel occlusion“, LVO), d. h. der proximalen Anteile der A. cerebri media bzw. des intrakraniellen Abschnitts der A. carotis interna, in Bezug auf die funktionelle Beeinträchtigung wirksam sind, gilt inzwischen als gesichert [10]. Die Ergebnisse großer Metaanalysen stellen dabei die Bedeutung des Faktors Zeit heraus, weil Behandlungsergebnisse desto besser erscheinen, je schneller die Therapie begonnen worden ist [11]. Im Zuge der Prozessoptimierung beschäftigen sich Krankenhäuser nun kontinuierlich mit einer Verkürzung der Zeit vom Eintreffen in der Notfallaufnahme bis zur Leistenpunktion. Diese „Door-to-groin-puncture“-Zeit wird als Qualitätsindikator innerhalb der Einrichtung angesehen und für überregionale Vergleiche herangezogen. Während der patientenbezogenen Vorbereitung der endovaskulären Therapie taucht im interdisziplinären Austausch zwangsläufig die Frage auf, ob bei Patienten mit einem Hauptstammverschluss und unmittelbar anstehender endovaskulärer Therapie vorab eine systemische Thrombolyse begonnen werden sollte. Die gegen ein solches „Bridging“-Konzept sprechenden theoretischen Überlegungen schließen v. a. die Beobachtung ein, dass die Chancen der Wiedereröffnung betroffener Gefäßabschnitte durch die Thrombolyse allein sehr gering sind [12] und die Thrombolyse zudem mit Komplikationen wie Blutungen und allergischen Reaktionen behaftet ist.

In einer umfangreichen Metaanalyse adressierten Katsanos et al. [13] nun Nutzen und Sicherheit des Bridging-Konzepts und bezogen hierfür 38 in den Jahren 2012–2019 veröffentlichte Beobachtungsstudien und Post-hoc-Analysen randomisierter Studien mit insgesamt 11.798 Patienten mit einem Hauptstammverschluss ein. Während 5191 Patienten (44 %) ausschließlich eine endovaskuläre Therapie erhielten, erfuhren 6607 der Patienten (56 %) entsprechend dem Bridging-Konzept eine systemische Thrombolyse, kombiniert mit der endovaskulären Therapie. Primärer Endpunkt war eine funktionelle Unabhängigkeit zum Zeitpunkt der Entlassung aus dem Krankenhaus bzw. 3 Monate nach dem Ereignis, definiert als mRS 0–2. Die Wahrscheinlichkeit, diesen Endpunkt zu erreichen, war bei Patienten mit angewandtem Bridging-Konzept gegenüber der alleinigen endovaskulären Therapie signifikant erhöht (OR 1,55; KI 1,26–1,791; *p* < 0,0001). Auch zeigte sich die Wahrscheinlichkeit einer erfolgreichen Rekanalisation unter Anwendung des Bridging-Konzepts signifikant erhöht (OR 1,22; KI 1,02–1,46; *p* = 0,03). Hinsichtlich der Sicherheit war das Bridging-Konzept im Vergleich zur alleinigen endovaskulären Therapie sogar mit einer geringeren Sterblichkeit assoziiert (OR 0,80; KI 0,66–0,97; *p* = 0,02). Die Rate an symptomatischen intrakraniellen Blutungen unterschied sich zwischen den beiden Gruppen dagegen nicht (OR 0,87; KI 0,61–1,25; *p* = 0,46).

Die Metaanalyse von Katsanos et al. (2019) zeigte unter Einbeziehung einer sehr großen Patientenzahl einen Vorteil des Bridging-Konzepts in Bezug auf das funktionelle Behandlungsergebnis bei gleichzeitig nichterhöhter Blutungsrate. Bei fehlenden Kontraindikationen, speziell Bedingungen, die *per se* mit einem erhöhten Blutungsrisiko assoziiert sind, sollte Patienten mit zugrunde liegendem Hauptstammverschluss eine systemische Thrombolyse daher nicht vorenthalten werden, auch wenn sich die endovaskuläre Therapie unmittelbar an den Beginn der Thrombolyse anschließt.

## Peri- und postprozedurales Management der Thrombektomie

### Originalpublikation

Schönenberger S, Hendén PL, Simonsen CZ et al (2019) Association of general anesthesia vs procedural sedation with functional outcome among patients with acute ischemic stroke undergoing thrombectomy: a systematic review and meta-analysis. JAMA 322:1283–1293

Im Zuge der Etablierung endovaskulärer Therapieverfahren bei Patienten mit Hauptstammverschlüssen rückten verschiedene Aspekte des periprozeduralen Managements in den Vordergrund. Frühzeitig entstand die Frage, ob der Eingriff in Allgemeinanästhesie oder lediglich Analgosedierung ohne Atemwegssicherung erfolgen soll. Erste Daten hierzu stammten aus retrospektiven Betrachtungen einzelner Studien (z. B. van den Berg et al., [14]), und es folgten Metaanalysen (z. B. Brinjikji et al. [15]), die einen Vorteil für die Analgosedierung bzw. sogar ein deutlich schlechteres Behandlungsergebnis im Zusammenhang mit der Allgemeinanästhesie beschrieben. Vielerorts hatten diese Daten Implikationen für die Behandlung mit dem scheinbar auf einer guten Datenbasis fußenden Handlungsdruck, Patienten mit Hauptstammverschlüssen vorzugsweise unter Anwendung einer Analgosedierung, nicht aber einer Allgemeinanästhesie, zu behandeln. Im interdisziplinären Setting erschienen hierdurch Konflikte vorprogrammiert, da auf der individuellen Patientenebene Faktoren, wie eine fehlende Kooperation bei aphasischen Störungen, eine ohnehin reduzierte Vigilanz bzw. eingeschränkte respiratorische Situation mit *per se* bestehender Indikation zur Atemwegssicherung oder auch erschwerte Lokalverhältnisse der hirnversorgenden Gefäße, die Eingriffe in alleiniger Sedierung nicht realistisch erschienen ließen. Von großer Relevanz waren daher solide, aus prospektiven Untersuchungen stammende Daten, die eine verlässliche Aussage zu den Vorzügen und möglichen Nachteilen beider Verfahren im Zusammenhang mit der endovaskulären Therapie erlauben.

In einer aktuellen Metaanalyse, die ausschließlich randomisierte Studien berücksichtigte, untersuchten Schönenberger et al. [16] den Effekt der Allgemeinanästhesie bzw. Analgosedierung bei Patienten, die aufgrund eines Hauptstammverschlusses und eines relevanten neurologischen Defizits (National Institutes of Health Stroke Scale (NIHSS) 10 oder mehr) eine endovaskuläre Therapie erhielten. Durch den Zusammenschluss der 3 randomisierten, endpunktverblindeten, im Zeitraum von 2016–2018 veröffentlichten Studien SIESTA (Deutschland), AnSTROKE (Schweden) und GOLIATH (Dänemark), konnten Daten von 368 Patienten ausgewertet werden, von denen 183 (49,7 %) eine Allgemeinanästhesie und 185 (50,3 %) eine Analgosedierung erhalten hatten. Primärer Endpunkt war der nach 3 Monaten mithilfe der mRS beurteilte funktionelle Zustand. Um Effekte möglichst breit abzubilden, wurden 15 sekundäre Endpunkte definiert, die neben klinischen Parametern (z. B. frühe, d. h. innerhalb von 24 h vorhandene Verbesserung der NIHSS) auch bildbasierte Merkmale (z. B. Rate an erfolgreichen Rekanalisationen) sowie ein Monitoring diverser Zeitintervalle während der Behandlung einbezogen. Im Ergebnis wiesen Patienten, bei denen die endovaskuläre Therapie in Allgemeinanästhesie erfolgte, einen mittleren mRS von 2,8 nach 3 Monaten auf, was signifikant besser war als der mittlere mRS von 3,2 in der Patientengruppe mit erfolgter Analgosedierung (OR 1,58; KI 1,09–2,29; *p* = 0,02). Konsekutiv erreichten signifikant mehr Patienten mit erfolgter Allgemeinanästhesie gegenüber der Analgosedierung einen guten funktionellen Zustand (mRS 0–2) nach 3 Monaten (49,2 gegenüber 35,1 %; *p* = 0,003). Hingegen zeigten die frühe neurologische Verbesserung wie auch die Sterblichkeit keine statistisch signifikanten Unterschiede zwischen beiden Gruppen (*p* = 0,22 bzw. *p* = 0,51). Hinsichtlich der bildbasierten Parameter war unter den Bedingungen der Allgemeinanästhesie bei 156 Patienten (85,2 %) eine erfolgreiche Rekanalisation möglich, wogegen diese nur bei 140 Patienten (75,5 %) mit erfolgter Analgosedierung gelang (*p* = 0,01). Erwartungsgemäß war die Rate an Hypotonien, definiert als mindestens 20 %ige Reduktion gegenüber dem Ausgangswert, bei 143 der Patienten (80,8 %) mit Allgemeinanästhesie und 95 Patienten (53,1 %) mit erfolgter Analgosedierung, und damit signifikant häufiger im Zusammenhang mit der Allgemeinanästhesie, nachzuvollziehen (*p* < 0,001). Hinsichtlich der behandlungsprozessbezogenen Merkmale unterschieden sich die Gruppen beispielsweise in der Door-to-groin-puncture-Zeit nur marginal (im Mittel 75 min unter den Bedingungen der Allgemeinanästhesie und 69 min im Fall der Analgosedierung), was jedoch statistische Signifikanz erreichte (*p* = 0,04).

Die Metaanalyse von Schönenberger et al. [16] kann als im interdisziplinären Schnittstellenbereich besonders bedeutsame Veröffentlichung angesehen werden, da sie zahlreiche Fragen zum periprozeduralen Management endovaskulärer Therapien auf der Grundlage ausschließlich randomisierter Studien beantwortet. Im Gegensatz zu früheren Metaanalysen, die überwiegend retrospektive Studien berücksichtigten, zeigt die Arbeit sogar einen Vorteil für die Allgemeinanästhesie im Hinblick auf den funktionellen Zustand 3 Monate nach dem Eingriff. Damit bekräftigt die Studie die vielerorts erfolgende Praxis einer patientenbezogenen Wahl des Verfahrens unter Berücksichtigung individueller Merkmale wie der Kooperation und Vigilanz des einzelnen Patienten. Gleichzeitig dürfte sich der zuvor mindestens gefühlte Handlungsdruck in Richtung einer ausschließlichen Anwendung der Analgosedierung mit nun verbesserter Datenbasis gänzlich auflösen.

### Originalpublikation

Fandler-Höfler S, Heschl S, Kneihsl M et al (2020) Ventilation time and prognosis after stroke thrombectomy: the shorter, the better! Eur J Neurol 27:849–855

Dass allein der Umstand der Allgemeinanästhesie mit naturgemäßer Beatmung nicht mit einem schlechteren Behandlungsergebnis bei der endovaskulären Therapie von Patienten mit Hauptstammverschlüssen assoziiert ist, haben Schönenberger et al. [16] zeigen können. Dennoch bleibt die Frage offen, ob sich hierbei zeitabhängige Effekte ergeben.

In einer monozentrischen Kohortenstudie untersuchten Fandler-Höfler et al. [17] den Zusammenhang zwischen der Beatmungsdauer und dem funktionellen, mit der mRS beurteilten Status 3 Monate nach dem Eingriff. Berücksichtigt wurden die Daten von 447 Patienten, die im Zeitraum von 2011–2019 in Graz (Österreich) eine endovaskuläre Therapie aufgrund eines Hauptstammverschlusses erhielten. Der Median der Beatmungsdauer betrug 3 h. Im Einzelnen wurden 258 Patienten (57,7 %) innerhalb von 6 h, 124 Patienten (27,7 %) innerhalb von 24 h und 65 Patienten (14,5 %) außerhalb eines Zeitfensters von 24 h extubiert. Als primärer Endpunkt wurde ein guter funktioneller Zustand (mRS 0–2) definiert, den 188 Patienten (42,6 %) erreichten. Patienten, die innerhalb von 6 h extubiert werden konnten, wiesen im Vergleich zu Patienten, bei denen dies erst im Zeitfenster zwischen 6 und 24 h gelang, eine höhere Wahrscheinlichkeit für einen guten funktionellen Zustand 3 Monate nach dem Eingriff auf (OR 2,4; KI 1,53–63,76; *p* < 0,001). Eine längere Beatmungsdauer war assoziiert mit einer höheren Rate an Pneumonien: 9,6 % bei Patienten mit erfolgter Extubation innerhalb von 6 h, 20,6 % bei Patienten mit einer Extubation innerhalb von 24 h und 27,7 % bei einer Extubation jenseits eines Zeitfensters von 24 h (*p* < 0,01). Erwartungsgemäß waren als Ursachen für die verspätete Extubation in einem Zeitfenster von mehr als 24 h schlaganfallspezifische Komplikationen wie ein Hirnödem und eine eingeschränkte Wachreaktion eingrenzbar. Interessanterweise war eine Extubation im Zeitfenster zwischen 6 und 24 h mit der Krankenhauseinweisung außerhalb der Kernarbeitszeit assoziiert (*p* < 0,001). Bei der Interpretation der Studienergebnisse ist zu berücksichtigen, dass Patienten mit erfolgter Extubation nach mehr als 24 h bereits in der Aufnahmesituation ein schwereres neurologisches Defizit (NIHSS 17 gegenüber 14 in der Gruppe mit früherer Extubation; *p* < 0,001) und die geringste Rate an erfolgreichen Rekanalisationen aufwiesen (76,6 % gegenüber 92,5 % in der Gruppe mit früherer Extubation; *p* = 0,001). Die erhöhte Wahrscheinlichkeit eines komplikationsreicheren Verlaufs und damit auch einer prolongierten Beatmung war demnach in der Gruppe mit verlängerter Beatmung bereits im frühen Behandlungsverlauf erkennbar, sodass Aussagen zum Kausalzusammenhang kaum möglich erscheinen.

Trotz ihrer methodischen Limitationen liefert die Studie von Fandler-Höfler et al. [17] Hinweise auf einen „dosisabhängigen“ Effekt in Bezug auf die Beatmungsdauer und das funktionelle Behandlungsergebnis nach der endovaskulären Therapie. Für den klinischen Alltag ergibt sich aus diesen Daten, dass die Extubation möglichst frühzeitig nach dem Eingriff erfolgen sollte. Die Beobachtung des statistischen Zusammenhangs einer verlängerten Beatmung mit dem Aufnahmezeitpunkt im Krankenhaus führt zur Empfehlung, dass eine Extubation (und wenn notwendig auch die Reintubation) unter kontrollierten Bedingungen zu jedem Zeitpunkt möglich sein sollte, wofür personelle und materielle Voraussetzungen der nachbetreuenden Abteilungen vorgehalten werden müssen.

### Originalpublikation

Anadani M, Orabi MY, Alawieh A et al (2019) Blood pressure and outcome after mechanical thrombectomy with successful revascularization. Stroke 50:2448–2454

Einen weiteren Aspekt des periprozeduralen Managements bei endovaskulären Therapieverfahren adressierend, beschäftigten sich jüngste Untersuchungen mit dem optimalen Zielkorridor für den Blutdruck. Im Gegensatz zur systemischen Thrombolyse, für die in der fachspezifischen Leitlinie die Empfehlung für eine obere Blutdruckgrenze von 185/110 mm Hg enthalten ist [18], fehlen für endovaskuläre Therapieverfahren bislang derart konkrete Handlungsempfehlungen. Theoretische Überlegungen zielen auf eine sinnvoll zu wählende Obergrenze ab, durch die während und nach der abrupten Wiedereröffnung des verschlossenen Hirngefäßes eine Einblutung in nachgeschaltete Gewebe vermieden werden soll. Andererseits finden sich Hinweise darauf, dass ein zu geringer Blutdruck bzw. im Besonderen ein gegenüber dem Ausgangswert eintretender Blutdruckabfall während des Eingriffs zu einem ungünstigeren Behandlungsergebnis führen [19].

In einer multizentrischen Kohortenstudie unter Einbeziehung von 10 Zentren in den USA, Deutschland, Frankreich und Großbritannien, untersuchten Anadani et al. [20] den Zusammenhang zwischen dem bei Aufnahme sowie innerhalb der ersten 24 h gemessenen Blutdruck und dem Behandlungsergebnis bei Patienten mit zugrunde liegendem Hauptstammverschluss und erfolgter endovaskulärer Therapie mit erfolgreicher Rekanalisation. Im Zeitraum von 2015 bis 2018 konnten 1245 Patienten eingeschlossen werden. Endpunkte waren der mithilfe der mRS beurteilte funktionelle Zustand 3 Monate nach dem Eingriff sowie die Rate an symptomatischen intrazerebralen Blutungen, die Sterblichkeit und die Rate an notwendig gewordenen dekompressiven Hemikraniektomien infolge der Ausbildung raumfordernder Infarkte oder Blutungen. Nach 3 Monaten erreichten 567 (49 %) der Patienten einen guten funktionellen Zustand (mRS 0–2). Das Erreichen dieses Endpunkts war assoziiert mit einer geringeren Wahrscheinlichkeit bei Vorliegen eines im Vergleich zu den übrigen Patienten erhöhten mittleren systolischen Blutdrucks (OR 0,86; KI 0,79–0,93; *p* < 0,001), eines erhöhten maximalen systolischen Blutdrucks (OR 0,90; KI 0,85–0,95; *p* < 0,001) und der Schwankungsbreite des systolischen Blutdrucks (OR 0,91; KI 0,86–0,96; *p* = 0,003), sodass diese Parameter einen inversen statistischen Zusammenhang aufwiesen. Ausgangspunkt weiterer Analysen war eine Stratifizierung des mittleren systolischen Blutdrucks in die Gruppen (a) weniger als 100 mm Hg, (b) 101–120 mm Hg, (c) 121–140 mm Hg, (d) 141–160 mm Hg und (e) mehr als 160 mm Hg. Patienten der Gruppen (d) und (e) mit einem mittleren systolischen Blutdruck von mehr als 140 mm Hg wiesen eine signifikant geringere Wahrscheinlichkeit für das Erreichen eines guten funktionellen Zustands auf (*p* < 0,05). Ein mittlerer systolischer Blutdruck von 141–160 mm Hg (Gruppe (d)) war mit einer erhöhten Sterblichkeit und ein mittlerer systolischer Blutdruck von 101–120 mm Hg (Gruppe (b)) mit einer geringeren Wahrscheinlichkeit für eine symptomatische intrazerebrale Blutung assoziiert (*p* < 0,05). Hinsichtlich der Notwendigkeit dekompressiver Hemikraniektomien fand sich eine größere Wahrscheinlichkeit bei Patienten mit einem mittleren systolischen Blutdruck von 141–160 mm Hg (Gruppe (d); *p* < 0,05) sowie bei Patienten mit mehr als 160 mm Hg (Gruppe (e)), wenngleich der Zusammenhang in der letztgenannten Gruppe aufgrund des großen Konfidenzintervalls nicht statistisch signifikant war. Obwohl randomisierte Studien hinsichtlich des optimalen Blutdruckbereichs im Zusammenhang mit endovaskulären Therapieverfahren beim Hirninfarkt weiter ausstehen, lieferte die Untersuchung von Anadani et al. [20] doch Hinweise darauf, dass mithilfe einer guten Blutdruckkontrolle sowohl das funktionelle Behandlungsergebnis im längerfristigen Verlauf als auch kurzfristig auftretende Komplikationen moduliert werden können. Für konkrete Empfehlungen sind allerdings randomisierte und gut kontrollierte Studien notwendig.

## Zusammenfassung und Ausblick

Auch im Jahr 2019/2020 wurden mehrere Studien mit Implikationen für die klinische Praxis veröffentlicht (Übersicht in Tab. 1). In einer randomisierten Studie zum Status epilepticus fanden sich bezüglich Wirksamkeit und Sicherheit keine statistisch signifikanten Unterschiede zwischen den Substanzen Valproat, Levetiracetam und Fosphenytoin, sodass sich keine Präferenz bei der Anwendung in der 2. Stufe der Statusbehandlung ergibt. Die Auswahl sollte sich somit an den individuellen Begleiterkrankungen, im Besonderen hinsichtlich der Organfunktionen von Leber und Nieren sowie etwaiger kardialer Grunderkrankungen, orientieren.

Für Patienten mit infratentoriellen, speziell zerebellär gelegenen Hirnblutungen konnte eine Metaanalyse auf der Basis von Beobachtungsstudien keinen Vorteil eines chirurgischen Eingriffs (Hämatomausräumung) in Bezug auf den funktionellen Status zeigen. Allerdings war der Eingriff mit einer geringeren Sterblichkeit assoziiert und erscheint somit im Einzelfall als Ultima-Ratio-Ansatz vertretbar. Obgleich es für eine abschließende Beurteilung des Nutzens der chirurgischen Therapie bei infratentoriellen Hirnblutungen der Durchführung randomisierter Studien bedarf, kann die referierte Studie doch als Hilfestellung bei der individuellen Beratung herangezogen werden und zu einer individualisierten Therapieplanung beitragen.

Hinsichtlich der systemischen Thrombolyse im Kontext mit endovaskulären Therapieverfahren zeigte eine umfangreiche Metaanalyse einen klaren Vorteil für das sich aus der Historie ergebende Bridging-Konzept, d. h. die schnellstmögliche Initiierung der Thrombolyse bei fehlenden Kontraindikationen auch vor dem geplanten endovaskulären Eingriff.

Eine aktuelle Metaanalyse des periprozeduralen Managements endovaskulärer Therapieverfahren des Hirninfarkts konnte – unter ausschließlicher Einbeziehung randomisierter Studien – die in den vergangenen Jahren angenommenen, nachteiligen Effekte der Allgemeinanästhesie in Bezug auf das funktionelle Behandlungsergebnis nicht bestätigen. Daher sollte sich die Wahl des Verfahrens derzeit an patientenbezogenen Faktoren orientieren. Die Beatmungsdauer sollte dennoch möglichst kurz gehalten werden, weil sich ein statistisch signifikanter, nachteiliger Zusammenhang zwischen der Beatmungsdauer und dem funktionellen Ergebnis abzeichnet. Randomisierte Studien zum optimalen Blutdruckbereich im zeitlichen Zusammenhang mit endovaskulären Therapieverfahren stehen weiter aus. Derartige Studien sind jedoch dringend notwendig, um konkrete Handlungsempfehlungen geben zu können, denn die vorhandenen Daten aus nichtrandomisierten Untersuchungen legen einen Effekt auf das Behandlungsergebnis nahe.
